# Evaluation of the relationship between gastroptosis and reflux in pediatric patients

**DOI:** 10.3389/fmed.2025.1543297

**Published:** 2025-05-12

**Authors:** Sevgi Ulusoy Tangul, Atilla Senayli

**Affiliations:** Department of Pediatric Surgery, Faculty of Medicine, Yozgat Bozok University, Yozgat, Türkiye

**Keywords:** gastroptosis, gastroesophageal reflux, alkaline reflux, pediatric, upper GI series

## Abstract

**Purpose:**

Gastroesophageal reflux (GER) is a common issue in childhood, characterized by the regurgitation of stomach contents into the esophagus. As a result, pain in the epigastric region, nausea, and vomiting may occur. Gastroptosis, on the other hand, is an anatomical anomaly defined by the downward displacement of the stomach into the pelvic region and is rarely reported in the literature. The overlapping symptoms of these two conditions suggest a potential relationship between them. This study aims to determine the relationship between patients diagnosed with GER and those found to have gastroptosis using contrast-enhanced esophagus, stomach, and duodenum X-ray [upper gastrointestinal (GI) series] among patients presenting to the Pediatric Surgery Clinic.

**Methods:**

The study included 64 patients aged 1–18 years who presented with chronic epigastric region pain, nausea, and vomiting suggestive of GER. The patients were divided into two groups: those diagnosed with gastroptosis (*n* = 36) and a control group with normal stomach positioning (*n* = 28). Gastroptosis was classified into three grades based on stomach positioning, and gastroptosis and control groups were compared regarding sociodemographic data, findings from upper GI series, and pH monitoring results.

**Results:**

The median age of the study group was 14 years (3–18 years), and the majority of the patients were female (28/36). Patients with gastroptosis had significantly higher rates of alkaline reflux (*p* = 0.037). Although the frequency of alkaline reflux increased as the degree of gastroptosis increased, no significant difference was observed in the rates of acid reflux between the control and gastroptosis groups.

**Conclusion:**

The incidence of alkaline reflux is higher in children with gastroptosis. This suggests relationship between anatomic changes in the stomach and alkaline reflux. This study contributes to the literature as one of the first to demonstrate a connection between gastroptosis and alkaline reflux in pediatric patients, contributing to the literature. Considering the serious complications associated with alkaline reflux, such as Barrett’s esophagus and esophageal cancer, it is recommended that children with gastroptosis be managed with conservative treatment and closely monitored.

## 1 Introduction

Gastroesophageal reflux (GER) is a pathology that develops as a result of the reflux of stomach contents into the esophagus, and is a frequently encountered condition in pediatric surgical practice, especially with complaints such as pain in the epigastric region, nausea, and vomiting. However, in some patients with chronic abdominal pain, GER may go undetected if appropriate diagnostic evaluations are not conducted (1). Upper gastrointestinal (GI) series [contrast-enhanced esophagus, stomach, and duodenum X-ray] are routinely used in children to assess the esophagus, stomach, and proximal small intestine, and to identify structural anomalies if present (2, 3). Although upper GI series are not necessary for diagnosing GER, they are known to be essential for detecting conditions that may predispose to reflux. Gastroptosis is one such condition.

Visceroptosis, refers to the prolapse of internal organs as a consequence of adopting an upright posture (4). When the literature is reviewed, gastroptosis, a variant of visceroptosis, first appears in articles written toward the end of the 1800s (5). In his article, Beilin states that visceroptosis was first described by Glénard in 1833 (4). Since then, visceroptosis has also been referred to as “Glenard’s disease.” When it involves the stomach, it is termed “gastroptosis” (4, 6). The diagnosis of gastroptosis is made based on an upper GI series performed in an upright position, where the stomach is observed to have shifted downward, and the greater curvature descends partially below the level of the iliac crest (4, 7–9). Although its exact cause is unknown, it is often attributed to factors such as relaxation, stretching, or decreased muscle tone (4, 10). However, based on existing literature, gastroptosis primarily affects women aged 20–50 and is often associated with low body weight and postural abnormalities (4, 7, 9, 11). In the past, gastroptosis was often diagnosed. However, gastroptosis is rarely reported today, and its true prevalence, especially in childhood, is unknown. A study conducted in adults in Japan reported that gastroptosis was seen in 12% of men and 43% of women, and most patients were underweight (12). Gastroptosis may also be associated with poor posture. While not life-threatening, it can lead to symptoms such as constipation, vomiting, and indigestion, and tenesmus, loss of appetite, nausea, and belching. Patients with gastroptosis may experience postprandial discomfort, nausea, and unease (7). It is considered more of a pathological condition than a disease and is classified as either hereditary or acquired. Gastroptosis is diagnosed through an upper GI series when the stomach is observed to have shifted downward, with the greater curvature descending below the level of the iliac crest while the antrum remains in its normal position (11). Although the exact etiology is unclear, it is attributed to factors such as abdominal wall laxity, weakened gastric mesenteric attachments, and reduced adipose tissue in the lesser omentum (10, 13). In the early 19th century, surgical treatment was the primary approach for gastroptosis (11). Today, invasive treatments have been largely replaced by prokinetic drugs, abdominal strengthening exercises, and symptomatic measures such as abdominal bandages (9, 10).

**Figure 1:**
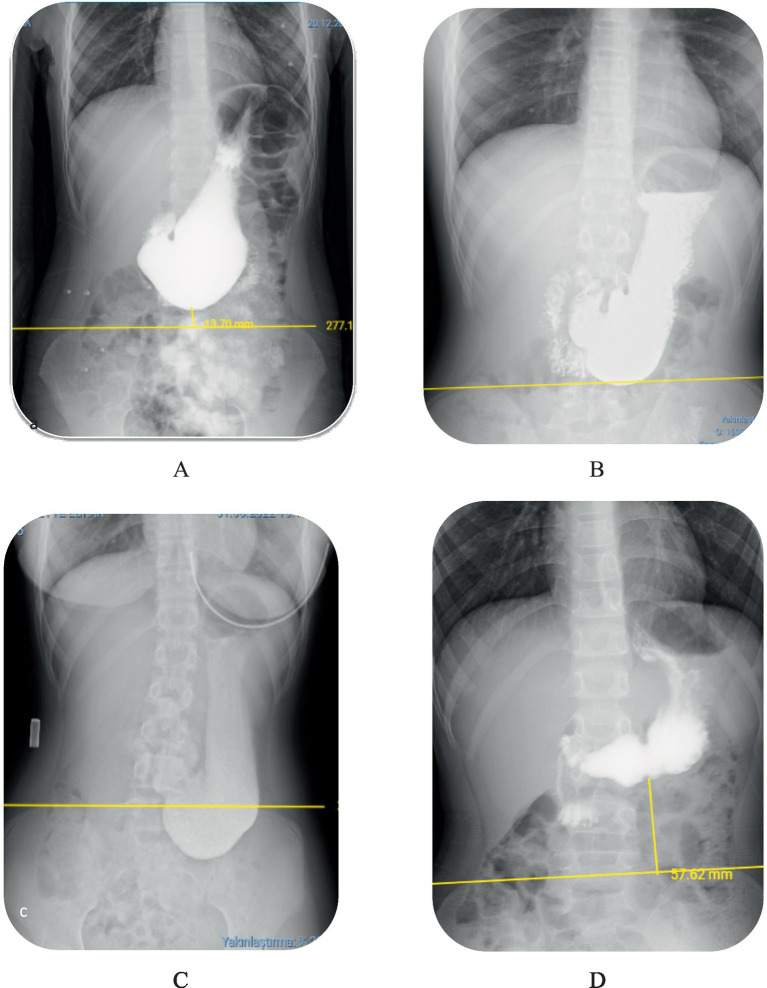
Degrees of gastroptosis. **(A)** Grade 1, **(B)** Grade 2, **(C)** Grade 3, **(D)** normal stomach (the yellow line represents the horizontal line drawn through the iliac crests).

Gastroptosis can be classified into three grades based on the distance between the greater curvature of the stomach and the iliac crest, as observed on an upper GI series. Although no studies in the literature include visual representations, some internet sources feature graphics similar to Steele’s article (14). This classification is based on an imaginary line drawn through the iliac crests:

Grade 1: The greater curvature approaches within 3 cm of the line (Figure 1A).

Grade 2: The greater curvature is at or directly on the line (Figure 1B).

Grade 3: The greater curvature is below the line (Figure 1C). Figure 1D shows the normal stomach in the upper GI series.

Gastroesophageal reflux (GER) is a condition generally acidic stomach contents into the esophagus due to decreased lower esophageal sphincter (LES) pressure and sphincter relaxation. This reflux can still occur at pH>4, but pH<4 is more problematic (15). Alkaline reflux, defined as the backflow of duodenal contents into the stomach and subsequently into the esophagus, is more commonly seen in adults as a secondary condition following partial gastrectomy, pyloroplasty, or cholecystectomy. Alkaline reflux, defined as the reflux of duodenal contents into the stomach and subsequently into the esophagus, commonly occurs as a secondary condition in adults following partial gastrectomy, pyloroplasty, or cholecystectomy, although the incidence of primary reflux without prior surgery has been increasingly reported (16, 17). However, it can also occur primarily in children and individuals without prior gastrointestinal surgery (18, 19). The literature gives different rates of alkaline reflux for the pediatric age group, such as 2–11.8% and acid reflux 88.2-98 % (20, 21). Lower esophageal pH > 7 is considered alkaline reflux (22). Alkaline reflux results from impaired motility in the antroduodenal region and dysfunction of the pyloric sphincter, causing bile to contact the esophageal mucosa, leading to endoscopic and histological pathologies (18, 23).

Both acid and alkaline reflux manifest clinically with symptoms such as bitter regurgitation, dyspepsia, vomiting, anemia, growth retardation, and coughing attacks. However, abdominal pain, particularly epigastric pain, is frequently observed in children (24). Upper GI series are commonly used in clinical practice to rule out other anatomical abnormalities that can cause in an increased incidence of GER. However, the definitive diagnostic procedure for GER remains 24-h pH monitoring (25). The literature mentions that approximately 2.3–15% of patients with chronic GERD may develop Barrett’s esophagus (1, 26). Barrett’s esophagus is characterized by the replacement of normal esophageal squamous cell epithelium with columnar metaplasia, and the first choice in its management is to use acid-suppressing drugs to reduce the underlying GERD symptoms (18, 26). Acid-suppressing drugs are used in the management of Barrett’s esophagus (BE) due to both acidic and alkaline reflux (27, 28).

Considering the overlapping symptoms of gastroptosis and GER, it raises the question of whether one is the cause or the consequence of the other. A review of the literature reveals no studies that evaluate the coexistence of these two pathologies or compare their relationship.

This study aims to retrospectively evaluate the upper GI series and 24-h pH monitoring results of patients admitted with gastrointestinal complaints lasting more than 3 months, such as chronic abdominal pain, nausea, and vomiting, and suspected GER. The goal is to determine the relationship between GER and gastroptosis.

## 2 Methods

The study received approval from the Bozok University Clinical Research Ethics Committee under protocol number 2017-KAEK-189_2022.10.27_05. Medical records of pediatric patients aged 1–18 years who were admitted to the Pediatric Surgery Clinic between 2021 and 2023 with gastrointestinal complaints lasting more than 3 months, such as chronic abdominal pain, nausea, bloating, and vomiting, were retrospectively reviewed. All patients underwent upper gastrointestinal (GI) series and 24-h pH monitoring with a high likelihood of gastroesophageal reflux (GER). Patients treated for conditions such as acute abdominal pain or acute appendicitis and those who did not undergo upper GI series or pH monitoring were excluded from the study.

The included patients were divided into a study group and a control group. The study group consisted of patients diagnosed with gastroptosis through upper GI series imaging and with available 24-h pH monitoring results. The control group included patients with normal gastric positioning on upper GI series and available 24-h pH monitoring results. Sociodemographic data (e.g., age, gender), gastroptosis grading based on the distance between the stomach and the iliac crest on the upper GI series, and 24-h pH monitoring data were recorded for all patients. All patients underwent 24-h esophageal pH monitoring using a dual-channel pH meter device. The pH probe was selected based on the patient’s height, ensuring a fixed distance of 5, 10, or 15 cm between Channel 1 and Channel 2. The probe’s position was determined according to anatomical reference points calculated from the patient’s height, and it was placed approximately 2.5 cm proximal to the lower esophageal sphincter (LES). Channel 1 was used to record pH values ≈2.5 cm from the LES, while Channel 2 was positioned to evaluate pH 5–10 cm from the LES. For both the study and control groups, the following parameters were compared in Channel 1 and Channel 2:

- Time with a pH ≤ 4 in upright and supine positions, and their means.
- Number of acidic reflux episodes within 24 h in upright, supine, and mean positions.
- Number of acidic reflux episodes lasting longer than 5 mins in upright, supine, and mean positions.
- Longest acidic reflux duration in upright, supine, and mean positions.
- Total number of ph ≤ 4 (The number of times the pH ≤ 4 in 24 h).
- Esophageal clearance time in upright, supine, and mean positions.
- Percentage of total alkaline time in the upright, supine, and Mean positions when pH > 7.
- Duration of alkaline periods in the upright, supine, and mean positions.
- Number of alkaline periods with pH > 7 lasting longer than 5 mins in the upright, supine and total alkaline time.

Data were analyzed with IBM SPSS V23. Compliance with normal distribution was examined using Shapiro–Wilk and Kolmogorov–Smirnov tests. A comparison of categorical variables according to groups was examined using Yates’ correction and Fisher’s exact test with Monte Carlo correction. One-way ANOVA was used to compare normally distributed data according to degrees, and multiple comparisons were examined with Tamhane’s T2 test and Bonferroni tests. A comparison of non-normally distributed data according to three or more groups was analyzed using the Kruskal-Wallis test, and multiple comparisons were made using the Dunn test. Relationships between non-normally distributed data were analyzed with Spearman’s rho correlation coefficient. Analysis results are presented as mean±s: deviation and median (min.-max.), and categorical data as frequency (percentage). Significance level was taken as *p* < 0.05.

## 3 Results

Among 112 patients who came to our pediatric surgery clinic with chronic upper gastrointestinal complaints and underwent upper GI series, data from 64 patients (57.14%) that underwent ambulatory pH meter examination were included in the study. The control group included 28 patients, while the study group consisted of 36 patients. The median age was 14 years (3–21) in the study group and 12 years (2 months–17) in the control group. The female-to-male ratio was 28/8 in the study group and 18/10 in the control group. Table 1 shows the distribution of female–male and age ratios by group. Table 2 shows gender comparisons according to the degree of gastroptosis, and Table 3 shows age comparisons according to the degree of gastroptosis.

**Table 1:** Gender and age ratios of gastroptosis and control groups.

Gender	Gastroptosis group	Control group	Total	Test statistics	*p*
Female	28 (77.8)	18 (64.3)	46 (71.9)	0.829	0.137^x^
Male	8 (22.2)	10 (35.7)	18 (28.1)		
Age (year)	14 (3–18)	12 (0–17)	13 (0–18)	544.500	0.586^y^

x Yates düzeltmesi.

y Mann-Whitney U testi; median (min–max), n(%).

**Table 2:** Comparison of gender according to the degrees of gastropitosis.

Gender	Grade 1	Grade 2	Grade 3	Test statistics	*p*
Female	9 (100)	9 (69.2)	10 (71.4)	3.506	0.189^x^
Male	0 (0)	4 (30.8)	4 (28.6)		

x Fisher’s Exact Test with Monte Carlo correction; n(%).

**Table 3:** Comparison of age according to the degrees of gastroptosis.

Degrees of gastroptosis	Mean ± SD (years)	Median (min–max; years)	Test statistics	*p*
Grade 1	13.33 ± 4.53	15 (6–18)	0.723	0.697^x^
Grade 2	11.92 ± 4.87	14 (3–17)		
Grade 3	11.57 ± 5.03	12.5 (3–18)		

x Kruskall Wallis H Test.

All statistically significant data are summarized in Table 4. When the gastroptosis group and the control group were compared based on 24-h pH monitoring results for Channel 1, a statistically significant difference was found in the total alkaline time (mean = 14.36, *p* = 0.037). No statistically significant differences were observed in other parameters (Table 5).

**Table 4:** Comparison results according to the degrees of gastropitosis group (summary table).

	Grade 1	Grade 2	Grade 3	Test statistics	*p*
Channel 1					
Supine total alkaline time (%; minutes)	15.78 ± 13.64^a^	38.16 ± 17.05^b^	34.41 ± 27.95^ab^	6.102	**0.008** ^y^
Mean total alkaline time (%; minutes)	20.29 ± 11.67^b^	39.91 ± 15.33^a^	33.47 ± 20.19^ab^	3.708	**0.035** ^y^
Supine alkaline period duration	51 (0–151)^a^	111 (17–643)^a^	133.5 (18–327)^a^	6.830	**0.033** ^x^
Supine long alkaline period count (>5 min)	2 (0–12)^a^	13 (1–30)^b^	7 (0–28)^ab^	7.080	**0.029** ^x^
Total alkaline time (minutes)	3 (0–19)^a^	19 (3–32)^b^	11.5 (0–36)^ab^	6.229	**0.044** ^x^
Channel 2					
Esophageal clearance time (upright; minutes)	0.93 ± 0.3^a^	0.69 ± 0.24^ab^	0.66 ± 0.2^b^	3.792	**0.033** ^y^
Mean esophageal clearance time (minutes)	1.1 (0.6–2.4)^a^	1 (0.7–1.3)^ab^	0.75 (0.4–1.2)^b^	7.303	**0.026** ^x^

x Kruskal Wallis H Test(*p* < 0.05).

y One Way Anova (*p* < 0.05).

There is no difference between groups with the same letter^a-b^. Channel 1: ≈2.5 cm from LES, Chanel 2: 5–10 cm from LES.

**Table 5:** Comparison of 24-h pH monitoring results in channel 1 between the gastroptosis group and the control group.

	Gastroptosis group (*n* = 36)				Control group (*n* = 28)				*p*
	Mean	Std. dev	Min	Max	Mean	Std. dev	Min	Max	
Upright total acidic reflux time (ph ≤ 4; minutes)	16.25	16.29	0.40	73.70	21.65	21.69	0.50	78.30	0.253
Supine total acidic reflux time (ph ≤ 4; minutes)	21.86	30.42	0.00	92.70	31.25	29.51	0.00	96.60	0.180
Mean total acidic reflux time (ph ≤ 4; minutes)	19.81	23.10	0.20	78.30	26.71	24.18	0.40	81.20	0.157
Upright acidic reflux episode count	110.39	112.20	6.00	383.00	111.71	95.05	4.00	441.00	0.368
Supine acidic reflux episode count	38.56	39.73	0.00	148.00	65.64	83.00	0.00	407.00	0.228
Mean acidic reflux episode count	148.39	131.41	6.00	447.00	177.36	139.82	7.00	566.00	0.242
Upright long acidic reflux episode count (>5 min)	3.39	5.75	0.00	30.00	4.68	6.78	0.00	27.00	0.413
Supine long acidic reflux episode count (>5 min)	6.78	9.12	0.00	29.00	9.43	11.20	0.00	53.00	0.301
Mean long acidic reflux episode count (>5 min)	10.17	13.09	0.00	43.00	14.11	13.99	0.00	57.00	0.146
Longest acidic reflux duration (upright)	13.19	16.51	1.00	81.10	24.04	51.63	1.20	269.50	0.561
Longest acidic reflux duration (supine)	32.89	53.11	0.00	276.00	55.85	80.70	0.00	363.30	0.196
Mean longest acidic reflux duration	35.22	52.16	1.60	276.00	59.99	83.66	1.30	363.30	0.144
Total number of ph ≤ 4	676.69	658.84	25.00	2804.00	631.89	461.57	91.00	1855.00	0.617
Esophageal clearance time (upright; minutes)	0.83	0.60	0.00	3.50	0.87	0.69	0.30	3.80	0.462
Esophageal clearance time (supine; minutes)	1.58	1.47	0.00	8.00	2.36	3.48	0.00	18.70	0.346
Mean esophageal clearance time (minutes)	1.17	0.60	0.00	2.60	1.41	1.38	0.40	8.00	0.734
Upright total alkaline time (%; minutes)	33.43	19.11	2.40	72.60	23.85	15.43	0.00	51.20	0.077
Supine total alkaline time (%; minutes)	31.11	22.71	0.00	88.00	25.52	25.59	0.00	95.30	0.176
Mean total alkaline time (%; minutes)	32.50	17.95	3.10	61.70	25.03	19.81	0.00	76.70	0.087
Upright alkaline period duration (minutes)	215.25	103.87	45.00	437.00	205.43	140.04	0.00	484.00	0.449
Supine alkaline period duration (minutes)	133.83	123.95	0.00	643.00	99.50	83.89	0.00	283.00	0.231
Mean alkaline period duration (minutes)	349.08	160.28	111.00	844.00	303.96	165.35	0.00	625.00	0.333
Upright long alkaline period count (>5 min)	5.03	6.55	0.00	29.00	2.18	2.96	0.00	12.00	0.069
Supine long alkaline period count (>5 min)	9.33	8.38	0.00	30.00	7.21	9.57	0.00	42.00	0.104
Total alkaline time (minutes)	14.36	11.34	0.00	36.00	9.39	10.55	0.00	45.00	0.037*

Channel 1: ≈2.5 cm from LES. *p < 0.05.

When the gastroptosis group and the control group were compared based on 24-h pH monitoring results for Channel 2, no statistically significant differences were observed (Table 6).

**Table 6:** Comparison of 24-h pH monitoring results in channel 2 between the gastroptosis group and the control group.

	Gastroptosis group (*n* = 36)				Control group (*n* = 28)				*p*
	Mean	Std. dev	Min	Max	Mean	Std. deviation	Min	Max	
Upright total acidic reflux time (ph ≤ 4; minutes)	24.80	22.71	2.50	105.00	23.84	21.46	0.20	80.10	0.973
Supine total acidic reflux time (ph ≤ 4; minutes)	25.22	27.92	0.20	81.30	26.39	27.30	0.00	84.30	0.914
Mean total acidic reflux time (ph ≤ 4; minutes)	24.49	22.29	1.60	73.90	25.54	23.57	0.90	80.80	0.844
Upright acidic reflux episode count	139.89	118.16	22.00	477.00	130.00	104.68	4.00	459.00	0.941
Supine acidic reflux episode count	71.75	70.16	3.00	307.00	74.21	83.63	0.00	376.00	0.761
Mean acidic reflux episode count	212.39	147.66	29.00	597.00	202.79	152.34	9.00	603.00	0.766
Upright long acidic reflux episode count (>5 min)	10.06	32.96	0.00	199.00	5.04	5.66	0.00	21.00	0.768
Supine long acidic reflux episode count (>5 min)	10.08	14.98	0.00	75.00	9.57	12.01	0.00	52.00	0.995
Mean long acidic reflux episode count (>5 min)	20.14	45.76	0.00	274.00	14.61	15.19	0.00	57.00	0.834
Longest acidic reflux duration (upright)	14.04	17.18	1.90	86.60	12.72	13.22	1.10	67.70	0.818
Longest acidic reflux duration (supine)	30.76	47.71	0.40	276.70	29.86	24.09	0.00	74.10	0.543
Mean longest acidic reflux duration	33.90	47.79	1.90	276.70	32.48	23.64	1.50	74.10	0.486
Total number of ph ≤ 4	605.69	489.22	100.00	1943.00	601.18	392.04	106.00	1707.00	0.626
Esophageal clearance time (upright; minutes)	0.74	0.26	0.30	1.40	0.78	0.47	0.00	1.80	0.902
Esophageal clearance time (supine; minutes)	1.44	1.03	0.30	5.30	1.57	1.18	0.00	5.20	0.485
Mean esophageal clearance time (minutes)	0.99	0.39	0.40	2.40	1.06	0.52	0.00	2.70	0.369
Upright total alkaline time (%; minutes)	28.08	17.48	3.30	60.10	26.27	18.60	0.00	73.70	0.641
Supine total alkaline time (%; minutes)	31.95	25.60	0.00	82.70	34.11	30.35	0.00	96.70	0.914
Mean total alkaline time (%; minutes)	30.48	19.62	2.50	70.10	30.57	24.02	0.00	80.30	0.844
Upright alkaline period duration (minutes)	175.39	91.59	14.00	471.00	173.79	136.75	0.00	476.00	0.372
Supine alkaline period duration (minutes)	97.39	71.18	0.00	266.00	77.18	72.29	0.00	340.00	0.176
Mean alkaline period duration (minutes)	272.78	119.04	52.00	563.00	250.96	152.72	0.00	552.00	0.444
Upright long alkaline period count (>5 min)	5.58	6.62	0.00	23.00	3.93	4.14	0.00	14.00	0.666
Supine long alkaline period count (>5 min)	8.53	7.44	0.00	29.00	8.50	9.48	0.00	44.00	0.640
Total alkaline time (minutes)	14.11	11.96	0.00	37.00	12.43	10.83	0.00	47.00	0.650

Chanel 2: 5–10 cm from LES.

When gastroptosis group, Grades 1, 2, and 3 were compared among themselves in channel 1 (Table 7); it was found that there was a statistical difference between the mean supine total alkaline time (%) values according to the gastroptosis degrees (*p* = 0.008). The mean value was 15.78 in Grade 1, 38.16 in Grade 2, and 34.41 in Grade 3. While there was no statistically significant difference between Grade 1 and Grade 3, there was a statistically significant difference between Grade 1 and Grade 2. There was no statistically significant difference between Grade 2 and Grade 3. It was found that there was a statistical difference between the mean total alkaline time (%) values according to the gastroptosis degrees (*p* = 0.035). The mean value was 20.29 in Grade 1, 39.91 in Grade 2, and 33.47 in Grade 3. While there was no statistically significant difference between Grade 1 and Grade 3, there was a statistically significant difference between Grade 1 and Grade 2. There was no statistically significant difference between Grade 2 and Grade 3. It was found that there was a statistical difference between the median supine alkaline period duration values according to the gastroptosis degrees (*p* = 0.033). The median value was 51 in Grade 1, 111 in Grade 2, and 133.5 in Grade 3. There was no statistically significant difference between Grade 1, Grade 2, and Grade 3. It was found that there was a statistical difference between the median supine long alkaline period count values according to the gastroptosis degrees (*p* = 0.029). While the median value was 2 in Grade 1, it was 13 in Grade 2 and 7 in Grade 3. While there was no statistically significant difference between Grade 1 and Grade 3, there was a statistically significant difference between Grade 1 and Grade 2. There was no statistically significant difference between Grade 2 and Grade 3. It was found that there was a statistically significant difference between the median mean total alkaline time values according to the gastroptosis degrees (*p* = 0.044). While the median value was 3 in Grade 1, it was 19 in Grade 2 and 11.5 in Grade 3. While there was no statistically significant difference between Grade 1 and Grade 3, there was a statistically significant difference between Grade 1 and Grade 2. There was no statistically significant difference between Grade 2 and Grade 3. When other parameters were compared, no significant difference was detected.

**Table 7:** Comparison results according to the degrees of gastropitosis group (channel 1).

	Grade 1	Grade 2	Grade 3	Test statistics	*p*
Age(year)	15 (6–18)	14 (3–17)	12.5 (3–18)	0.723	0.697^x^
Upright total acidic reflux time (ph ≤ 4; minutes)	12.8 (0.4–39.5)	9.6 (0.9–38.1)	10.35 (1.3–73.7)	1.289	0.525^x^
Supine total acidic reflux time (ph ≤ 4; minutes)	2.5 (0.2–92.7)	4.9 (0–86)	6.2 (0–85.6)	0.982	0.612^x^
Mean total acidic reflux time (ph ≤ 4; minutes)	11.7 (0.2–73.2)	7.9 (0.5–55.4)	9.05 (0.8–78.3)	0.527	0.769^x^
Upright acidic reflux episode count	106 (6–334)	42 (6–254)	60 (17–383)	3.703	0.157^x^
Supine acidic reflux episode count	8 (0–83)	32 (0–144)	39.5 (1–148)	3.755	0.153^x^
Mean acidic reflux episode count	173 (6–417)	70 (12–298)	109.5 (18–447)	1.823	0.402^x^
Upright long acidic reflux episode count (>5 min)	3 (0–12)	0 (0–12)	1.5 (0–30)	2.408	0.300^x^
Supine long acidic reflux episode count (>5 min)	1 (0–23)	3 (0–24)	2.5 (0–29)	0.816	0.665^x^
Mean long acidic reflux episode count (>5 min)	3 (0–35)	4.1 (0–36)	4 (0–43)	0.067	0.967^x^
Longest acidic reflux duration (upright)	11.2 (1.6–43.5)	4.9 (1–51.3)	6.55 (2.1–81.1)	1.498	0.473^x^
Longest acidic reflux duration (supine)	5 (0–276)	12.2 (0–89.9)	7.5 (0.3–133.4)	0.882	0.643^x^
Mean longest acidic reflux duration	17 (1.6–276)	12.2 (2–89.9)	8.05 (2.1–133.4)	0.302	0.860^x^
Total number of ph ≤ 4	518 (71–2014)	484 (147–1860)	364.5 (25–2,804)	0.141	0.932^x^
Esophageal clearance time (upright; minutes)	0.9 (0–1)	0.7 (0–1.6)	0.75 (0–3.5)	0.286	0.867^x^
Esophageal clearance time (supine; minutes)	1 (0–8)	1.4 (0.7–4.1)	1 (0.2–4.2)	2.858	0.240^x^
Mean esophageal clearance time (minutes)	1 (0–2)	1.2 (0.9–2.1)	1 (0–2.6)	2.948	0.229^x^
Upright total alkaline time (%; minutes)	23.82 ± 10.64	41.8 ± 19.93	31.83 ± 20.26	2.664	0.085^y^
Supine total alkaline time (%; minutes)	15.78 ± 13.64^a^	38.16 ± 17.05^b^	34.41 ± 27.95^ab^	6.102	**0.008** ^y^
Mean total alkaline time (%; minutes)	20.29 ± 11.67^b^	39.91 ± 15.33^a^	33.47 ± 20.19^ab^	3.708	**0.035** ^y^
Upright alkaline period duration (minutes)	252.11 ± 112.46	209.23 ± 114.81	197.14 ± 88.12	0.792	0.462^y^
Supine alkaline period duration (minutes)	51 (0–151)^a^	111 (17–643)^a^	133.5 (18–327)^a^	6.830	**0.033** ^x^
Mean alkaline period duration (minutes)	310.56 ± 141.1	393.54 ± 211.41	332.57 ± 111.17	0.612	0.553^y^
Upright long alkaline period count (>5 min)	1 (0–15)	4 (0–19)	2.5 (0–29)	1.887	0.389^x^
Supine long alkaline period count (>5 min)	2 (0–12)^a^	13 (1–30)^b^	7 (0–28)^ab^	7.080	**0.029** ^x^
Total alkaline time (minutes)	3 (0–19)^a^	19 (3–32)^b^	11.5 (0–36)^ab^	6.229	**0.044** ^x^

x Kruskal Wallis H Test (*p* < 0.05).

y One Way Anova (*p* < 0.05).

There is no difference between groups with the same letter^a-b^. Channel 1: ≈2.5 cm from LES. Values with *p* < 0.05 are shown in bold.

When gastroptosis group, Grades 1, 2, and 3 were compared among themselves in channel 2 (Table 8); it was found that there was a statistically significant difference between the mean esophageal clearance time (upright) values according to the degrees of gastroptosis (*p* = 0.033). While the mean value was 0.93 in Grade 1, it was obtained as 0.69 in Grade 2 and 0.66 in Grade 3. While there was no statistically significant difference between Grade 1 and Grade 2, there was a statistically significant difference between Grade 1 and Grade 3. There was no statistically significant difference between Grade 2 and Grade 3. It was found that there was a statistically significant difference between the Mean esophageal clearance time according to the degrees of gastroptosis (*p* = 0.026). While the median value was 1.1 in Grade 1, it was obtained as 1 in Grade 2 and 0.75 in Grade 3. While there was no statistically significant difference between Grade 1 and Grade 2, there was a statistically significant difference between Grade 1 and Grade 3. There was no statistically significant difference between Grade 2 and Grade 3. When other parameters were compared, no significant difference was detected.

**Table 8:** Comparison results according to the degrees of gastropitosis group (channel 2).

	Grade 1	Grade 2	Grade 3	Test statistics	*p*
Age(year)	15 (6–18)	14 (3–17)	12.5 (3–18)	0.723	0.697^x^
Upright total acidic reflux time (ph ≤ 4; minutes)	26.7 (2.9–105)	14.3 (3.3–64.9)	13.4 (2.5–63.1)	0.755	0.686^x^
Supine total acidic reflux time (ph ≤ 4; minutes)	15.2 (0.2–64.1)	10.3 (1.8–74.7)	8.4 (1.1–81.3)	0.573	0.751^x^
Mean total acidic reflux time (ph ≤ 4; minutes)	15.8 (1.6–52.4)	11.8 (2.4–73.9)	10.05 (1.7–70.3)	0.060	0.970^x^
Upright acidic reflux episode count	97 (26–337)	72 (34–253)	114.5 (22–477)	0.885	0.642^x^
Supine acidic reflux episode count	18 (3–107)	53 (12–307)	43 (16–165)	3.622	0.164^x^
Mean acidic reflux episode count	175.89 ± 138.91	201.92 ± 114.53	245.57 ± 180.28	0.648	0.530^y^
Upright long acidic reflux episode count (>5 min)	6 (0–17)	2 (0–199)	2 (0–27)	2.000	0.368^x^
Supine long acidic reflux episode count (>5 min)	4 (0–14)	5 (0–75)	3 (0–32)	1.754	0.416^x^
Mean long acidic reflux episode count (>5 min)	9 (0–31)	6 (0–274)	5 (0–51)	0.551	0.759^x^
Longest acidic reflux duration (upright)	11.8 (2.5–50.2)	6.9 (2.3–34.3)	8.3 (1.9–86.6)	2.600	0.273^x^
Longest acidic reflux duration (supine)	21.8 (0.4–276.7)	12.2 (3.9–71.6)	8.25 (1.2–73.4)	0.376	0.829^x^
Mean longest acidic reflux duration	34.7 (2.5–276.7)	12.2 (3.9–71.6)	10.55 (1.9–86.6)	1.479	0.477^x^
Total number of ph ≤ 4	355 (164–1,549)	360 (172–1,494)	497.5 (100–1943)	0.219	0.896^x^
Esophageal clearance time (upright; minutes)	0.93 ± 0.3^a^	0.69 ± 0.24^ab^	0.66 ± 0.2^b^	3.792	**0.033** ^y^
Esophageal clearance time (supine; minutes)	1.3 (0.3–5.3)	1.3 (0.8–2.1)	1.05 (0.3–3.1)	2.510	0.285^x^
Mean esophageal clearance time (minutes)	1.1 (0.6–2.4)^a^	1 (0.7–1.3) ^ab^	0.75 (0.4–1.2)^b^	7.303	**0.026** ^x^
Upright total alkaline time (%; minutes)	29.62 ± 19.73	30.49 ± 18.1	24.84 ± 16.18	0.386	0.683^y^
Supine total alkaline time (%; minutes)	12.5 (0–67.1)	35.1 (6.5–64.7)	42.65 (1.8–82.7)	2.565	0.277^x^
Mean total alkaline time (%; minutes)	14.9 (6.4–54.7)	33.5 (6.8–51.8)	35.65 (2.5–70.1)	0.236	0.889^x^
Upright alkaline period duration (minutes)	212.44 ± 113.37	151.08 ± 74.05	174.14 ± 89.83	1.210	0.311^y^
Supine alkaline period duration (minutes)	60.22 ± 52.33	109.38 ± 67.68	110.14 ± 80.33	1.702	0.198^y^
Mean alkaline period duration (minutes)	272.67 ± 128.27	260.46 ± 104.1	284.29 ± 133.14	0.128	0.880^y^
Upright long alkaline period count (>5 min)	3 (0–19)	5 (0–23)	3.5 (0–20)	0.217	0.897^x^
Supine long alkaline period count (>5 min)	3 (0–16)	11 (2–18)	7.5 (0–29)	3.500	0.174^x^
Total alkaline time (minutes)	10.11 ± 10.55	15.92 ± 12	15 ± 12.98	0.679	0.514^y^

x Kruskal Wallis H Test (*p* < 0.05).

y One Way Anova (*p* < 0.05).

There is no difference between groups with the same letter^a-b^. Chanel 2: 5–10 cm from LES.

When the correlation between variables and degrees of gastroptosis was examined, there was a statistically significant positive weak correlation between the degree of gastroptosis and the Supine reflux episode count (*r* = 0.263; *p* = 0.026). A statistically significant positive weak correlation exists between the degree of gastroptosis and the supine total alkaline time (%; *r* = 0.255; *p* = 0.031). A statistically significant positive weak correlation exists between the degree of gastroptosis and the supine alkaline period duration (*r* = 0.289; *p* = 0.014). No significant correlation was obtained between the degree and other variables (*p* > 0.05; Table 9).

**Table 9:** Correlation between variables and degrees of gastroptosis.

	Degrees of gastroptosis	
	*r*	*p*
Upright total acidic reflux time (ph ≤ 4; minutes)	−0.034	0.776
Supine total acidic reflux time (ph ≤ 4; minutes)	0.120	0.314
Mean total acidic reflux time (ph ≤ 4; minutes)	0.016	0.892
Upright acidic reflux episode count	−0.002	0.987
Supine acidic reflux episode count	**0.263**	**0.026**
Mean acidic reflux episode count	0.066	0.582
Upright long acidic reflux episode count (>5 min)	−0.061	0.609
Supine long acidic reflux episode count (>5 min)	0.062	0.604
Mean long acidic reflux episode count (>5 min)	−0.018	0.878
Longest acidic reflux duration (upright)	−0.103	0.388
Longest acidic reflux duration (supine)	0.022	0.857
Mean longest acidic reflux duration	−0.130	0.275
Total number of ph ≤ 4	0.027	0.820
Esophageal clearance time (upright; minutes)	−0.118	0.323
Esophageal clearance time (supine; minutes)	−0.170	0.153
Mean esophageal clearance time (minutes)	−0.221	0.062
Upright total alkaline time (%; minutes)	−0.019	0.877
Supine total alkaline time (%; minutes)	**0.255**	**0.031**
Mean total alkaline time (%; minutes)	0.167	0.160
Upright alkaline period duration (minutes)	−0.106	0.375
Supine alkaline period duration (minutes)	**0.289**	**0.014**
Mean alkaline period duration (minutes)	0.076	0.523
Upright long alkaline period count (>5 min)	0.049	0.682
Supine long alkaline period count (>5 min)	0.222	0.061
Total alkaline time (minutes)	0.159	0.183

r, Spearman’s rho correlation coefficient. Values with *p* < 0.05 are shown in bold.

## 4 Discussion

The results of this study showed that alkaline reflux was more prevalent in the patient group compared to the control group. Furthermore, the rate of alkaline reflux increased with the severity of gastroptosis. The absence of a significant difference in acid reflux rates between the patient and control groups, combined with the higher prevalence of alkaline reflux, suggests that preventing gastroptosis may reduce the harm caused by alkaline reflux.

The most common symptoms of gastroptosis are nonspecific and include epigastric pain, nausea, vomiting, bloating, and early satiety, particularly exacerbated by an upright posture or after meals (6, 10). Although our study did not specifically evaluate the correlation between these symptoms and existing literature, we found that the primary reasons for hospital admission in the patient group were consistent with these symptoms, particularly epigastric pain, nausea, vomiting, bloating, and early satiety after meals and in upright positions.

A study conducted in Japan found that dispeptic symptoms, such as ulcer-like dyspepsia in women and reflux-like dyspepsia in men, were less frequent in individuals with gastroptosis compared to controls without the condition. The authors suggested that gastroptosis might act as a protective factor rather than a disease (13). Although this study suggests that gastroptosis may have a protective effect in preventing dyspeptic symptoms, it appears that this conclusion was based more on patient-reported symptoms rather than evidence of a causal relationship. The absence of concrete studies demonstrating this relationship in the literature highlights that the findings of our study may be the first to provide such evidence.

In a 2012 study by Lincoln O. Diniz and colleagues, upper GI fluoroscopy and esophagograms of children with eosinophilic esophagitis were examined, and GER was identified as the most common cause of eosinophilic esophagitis. However, the study did not include evaluations of the stomach or duodenum (29). In another study evaluating upper GI series of children presenting to the pediatric clinic with nonspecific symptoms such as abdominal pain, growth retardation, and vomiting, the most common indications were non-bilious vomiting, GER symptoms, and abdominal pain (2). Among 720 patients who underwent upper GI series, 115 cases had positive findings that could cause these symptoms. Of these, 78 (68%) were associated with GER symptoms and signs (2). However, even in such a comprehensive study, gastroptosis was not evaluated as a cause of GER, and no data was found.

Hurst noted that while hypersthenic gastric structures are well-suited for efficient digestion, they may increase the risk of duodenal ulcers. In contrast, hyposthenic gastric structures were associated with a predisposition to hypochlorhydria, gastritis, achlorhydria, carcinoma, and pernicious anemia (30). Faber, in 1977, observed that the position of the stomach is correlated with chest width and is generally lower in women compared to men (31). In the same year, Conran noted that a low stomach position was generally associated with a tall, slender physique, particularly in men, and that both hyperacidity and hypoacidity were more common than normal in gastroptosis. However, he did not find any correlation between stomach position and the presence of gastric or duodenal ulcers (32). These studies appear to be among the earliest records concerning gastroptosis and gastric contents; however, to date, no study has confirmed these findings using quantitative data and measurements. This highlights that our study may be the first to demonstrate, with numerical evidence, the higher prevalence of reflux, particularly alkaline reflux, in patients with gastroptosis. Moreover, the fact that this finding was observed in children makes our study even more significant.

Barrett’s esophagus is a precancerous condition characterized by columnar metaplasia of the esophageal mucosa, developing secondary to chronic gastroesophageal reflux. The first step in treatment is acid-suppressive therapy aimed at reducing reflux symptoms (26). Although acid reflux is widely recognized as more influential in the development of Barrett’s esophagus (18, 19, 26), experimental studies suggest that alkaline reflux may also contribute to the development of esophageal adenocarcinoma. Clinical data show that patients with Barrett’s esophagus have significantly elevated bile acid levels, supporting the role of alkaline reflux in malignant progression (33–36). In the literature, an experimental study conducted in rats using the esophagogastroduodenal anastomosis (EGDA) model to induce chronic esophageal inflammation demonstrated that not only gastric acid, but also alkaline duodenal contents containing bile salts and active pancreatic enzymes may contribute to the development of Barrett’s metaplasia (37).

We believe that the undeniable status of alkaline reflux, which we have shown to be highly likely to develop in a pathology easily recognized in the upper GI series, such as gastroptosis, should not be ignored. If gastroptosis is detected by evaluating the upper GI series, alkaline reflux will be statistically much more likely than acid reflux.

## 5 Conclusion

Our study shows that alkaline reflux is more common in gastroptosis cases and its incidenc increases with the severity of gastroptosis. This finding suggests a possible relationship between the anatomical displacement of the stomach and the pathophysiology of alkaline reflux. Although it is impossible to prevent the development of gastroptosis completely, our study has shown which issues should be considered in these patients with conservative treatments and close follow-up to prevent complications that may develop with possible alkaline reflux. To our knowledge, our study is the first to describe this relationship. In this respect, it fills a gap in the literature and provides a basis for future research in this area.

## Data Availability

The original contributions presented in the study are included in the article/supplementary material, further inquiries can be directed to the corresponding author.
